# Passive heating-induced changes in muscle contractile function are not further augmented by prolonged exposure in young males experiencing moderate thermal stress

**DOI:** 10.3389/fphys.2024.1356488

**Published:** 2024-02-27

**Authors:** Viktorija Treigyte, Thomas Chaillou, Nerijus Eimantas, Tomas Venckunas, Marius Brazaitis

**Affiliations:** ^1^ Sports Science and Innovation Institute, Lithuanian Sports University, Kaunas, Lithuania; ^2^ School of Health Sciences, Örebro University, Örebro, Sweden

**Keywords:** hot-water immersion, muscle contractility, muscle fatigue, temperature, contractile properties, central activation, voluntary muscle activation, cardiovascular response

## Abstract

**Background:** We investigated the impact of 1) passive heating (PH) induced by single and intermittent/prolonged hot-water immersion (HWI) and 2) the duration of PH, on muscle contractile function under the unfatigued state, and during the development of muscle fatigue.

**Methods:** Twelve young males volunteered for this study consisting of two phases: single phase (SP) followed by intermittent/prolonged phase (IPP), with both phases including two conditions (i.e., four trials in total) performed randomly: control passive sitting (CON) and HWI (44–45°C; water up to the waist level). SP-HWI included one continuous 45-min bath (from 15 to 60 min). IPP-HWI included an initial 45-min bath (from 15 to 60 min) followed by eight additional 15-min baths interspaced with 15-min breaks at room temperature between 75 and 300 min. Intramuscular (Tmu; measured in the *vastus lateralis* muscle) and rectal (Trec) temperatures were determined. Neuromuscular testing (performed in the knee extensors and flexors) was performed at baseline and 60 min later during SP, and at baseline, 60, 90, 150 and 300 min after baseline during IPP. A fatiguing protocol (100 electrical stimulations of the knee extensors) was performed after the last neuromuscular testing of each trial.

**Results:** HWI increased Tmu and Trec to 38°C–38.5°C (*p* < 0.05) during both SP and IPP. Under the unfatigued state, HWI did not affect electrically induced torques at 20 Hz (P20) and 100 Hz (P100). However, it induced a shift towards a faster contractile profile during both SP and IPP, as evidenced by a decreased P20/P100 ratio (*p* < 0.05) and an improved muscle relaxation (i.e., reduced half-relaxation time and increased rate of torque relaxation; *p* < 0.05). Despite a reduced voluntary activation (i.e., −2.63% ± 4.19% after SP-HWI and −5.73% ± 4.31% after IPP-HWI; condition effect: *p* < 0.001), HWI did not impair maximal isokinetic and isometric contraction torques. During the fatiguing protocol, fatigue index and the changes in muscle contractile properties were larger after HWI than CON conditions (*p* < 0.05). Finally, none of these parameters were significantly affected by the heating duration.

**Conclusion:** PH induces changes in muscle contractile function which are not augmented by prolonged exposure when thermal stress is moderate.

## 1 Introduction

Heat therapy is regularly used for clinical purposes, and several benefits have been proposed, including improvements of endothelial function, cerebral blood flow, and glycemic control (Hunt et al., 2019). Athletes commonly apply passive heating (PH) such as hot-water immersion (HWI) to treat musculoskeletal injuries (McGorm et al., 2018), and although the advantages remain to be clearly demonstrated, this method is widely utilized to optimize post-exercise recovery (Chaillou et al., 2022). PH can also promote heat acclimation, which is essential for athletes and military personnel exercising in hot and humid conditions (Chaillou et al., 2022; Periard et al., 2022). In contrast, prolonged exposure to severe heat stress, combined or not with exercise, can lead to hyperthermia, which compromises physical capacity due to perturbations in the cardiovascular and central nervous systems, and skeletal muscle function (Periard et al., 2021).

Acute physiological responses to PH include increases in heart rate and energy expenditure, vasodilatation and enhanced cutaneous/muscle blood flow, and a rise in core, skin and intramuscular temperatures (Amin et al., 2022; Eimantas et al., 2022b). It is well-established that acute increase in intramuscular temperature (Tmu) induced by PH influences muscle contractile function (Rodrigues et al., 2022). Numerous studies have reported that PH improves muscle contractile properties during electrically evoked contraction (i.e., time to peak torque, rate of force development and half-relaxation time) (Davies et al., 1982; Rodrigues et al., 2021; Baranauskiene et al., 2023; Rodrigues et al., 2023). Tetanic force during electrically-evoked contraction is however not enhanced by PH at low frequency (10–20 Hz) (Davies et al., 1982; Periard et al., 2014b; Brazaitis et al., 2019; Baranauskiene et al., 2023), while it is either increased (Brazaitis et al., 2011) or not affected (Periard et al., 2014b; Brazaitis et al., 2019; Baranauskiene et al., 2023) at high frequency (50–100 Hz). Several studies have shown that maximal voluntary isometric contraction (MVIC) force is unaffected by PH (Davies et al., 1982; Stanley et al., 1994; Brazaitis et al., 2011; Brazaitis et al., 2019), while others have found a reduced MVIC force when rectal temperature (Trec) surpassed 39°C (Morrison et al., 2004; Thomas et al., 2006; Periard et al., 2014a). This heating-mediated impairment of MVIC force is usually ascribed to compromised voluntary activation (rather than to increased Tmu), indicative of a hyperthermia-induced failure in central neural drive to working muscles (Morrison et al., 2004; Thomas et al., 2006; Periard et al., 2014a). Following HWI of the lower limbs for 45 min at 44°C, peak isokinetic force of the knee extensors appears to be unchanged at low to high movement velocities (30°–400°/s) (Stanley et al., 1994), while others observed an increased isokinetic force during a maximal cycling sprint at 95 revolutions/min (Sargeant, 1987). Finally, PH leads to an accelerated force decline during sustained MVIC (Morrison et al., 2004; Brazaitis et al., 2012) and during repeated electrical stimulations (Brazaitis et al., 2011), and both central and peripheral mechanisms could be implicated into this impaired fatigue resistance. Altogether, PH-induced increase in Tmu seems to temporarily convert muscle contractile profile towards a faster one. To date, the effects of PH on force production during involuntary and voluntary contractions are equivocal, probably due to methodological differences in the heating protocols (method, temperature, duration, partial or whole-body exposure, etc.) and in the neuromuscular testing, and to divergent physiological responses to PH among the studied cohorts (e.g., magnitude of the changes in Tmu and Trec).

Heat stress can be experienced over prolonged periods in activities requiring muscle contractions (exercise, occupational activities, etc.), and this will be more severe and encountered increasingly more frequently with recent global warming and climate change (Moda et al., 2019). The majority of the studies focusing on muscle contractile function have used HWI protocols lasting up to 45 min (44°C–46°C) (Edwards et al., 1972; Davies and Young, 1983; Sargeant, 1987; Stanley et al., 1994; Brazaitis et al., 2011), while some recent studies have developed single HWI protocols lasting up to 90 min (42°C–43°C) (Rodrigues et al., 2021; Baranauskiene et al., 2023; Rodrigues et al., 2023). To determine whether the duration of PH *per se* influences muscle contractile function, it is essential to stabilize Tmu and Trec over prolonged periods. This will unlikely happen using prolonged and single HWI in regular lab conditions (i.e., ambient temperature of 20°C–24°C) (Baranauskiene et al., 2023) because whole body temperature (especially Trec) will gradually rise over time due to body heat accumulation. To prevent this, one alternate option is to apply intermittent HWI.

Some studies have provided evidence that heat stress-induced increase in Trec alters motor center control in the brain (Low et al., 2005; Baranauskiene et al., 2023), and that heat-related illness such as heat exhaustion is aggravated by the duration of heat stress (Periard et al., 2021). Thus, it can be hypothesized that prolonged PH and the associated prolonged elevation of Trec will result in a larger impairment of voluntary activation (compared with shorter exposure), leading to a marked reduction of MVIC force. Since Tmu is a primary factor determining muscle contractile properties (Davies and Young, 1983; Rodrigues et al., 2022), it is conceivable that the duration of PH will not affect these properties during electrically evoked contraction under the unfatigued state. However, high Tmu could increase ATP utilization and energy cost (Ball, 2021), and long-term heat stress upregulates some genes involved in ATP synthesis (Goto et al., 2011), potentially due to muscle energy depletion induced by repeated heat exposure. Therefore, we hypothesize that prolonged elevation of Tmu will exacerbate the decline of force during repeated electrical stimulations. To test these hypotheses, this study aimed to investigate the impact of 1) PH induced by single and intermittent/prolonged HWI and 2) the duration of PH, on muscle contractile function under the unfatigued state, and during the development of muscle fatigue. Under the unfatigued state, muscle force production was determined during involuntary contractions, and during voluntary isometric and isokinetic contractions, and muscle contractile properties were assessed during involuntary contractions. Muscle force and contractile properties were analyzed during development of fatigue resulting from electrically induced contractions.

## 2 Material and methods

This work is a follow-up to the recently published study (Treigyte et al., 2023), which investigated the impact of moderate muscle cooling induced by single and intermittent/prolonged cold-water immersions (CWI) on muscle force production and muscle contractility under the unfatigued state and during the development of fatigue. In this recent study, four experimental trials were performed: two trials including CWI (single phase and prolonged/intermittent phase) and two control trials including passive sitting (single phase and prolonged/intermittent phase). To answer the specific aims of the current study, data collected during the two control trials were also used. In addition, most of the information related to the participants, physiological measurements, neuromuscular testing, fatiguing protocol and data analysis is similar to that described in the previous article.

### 2.1 Rationale of the HWI protocols

**FIGURE 1:**
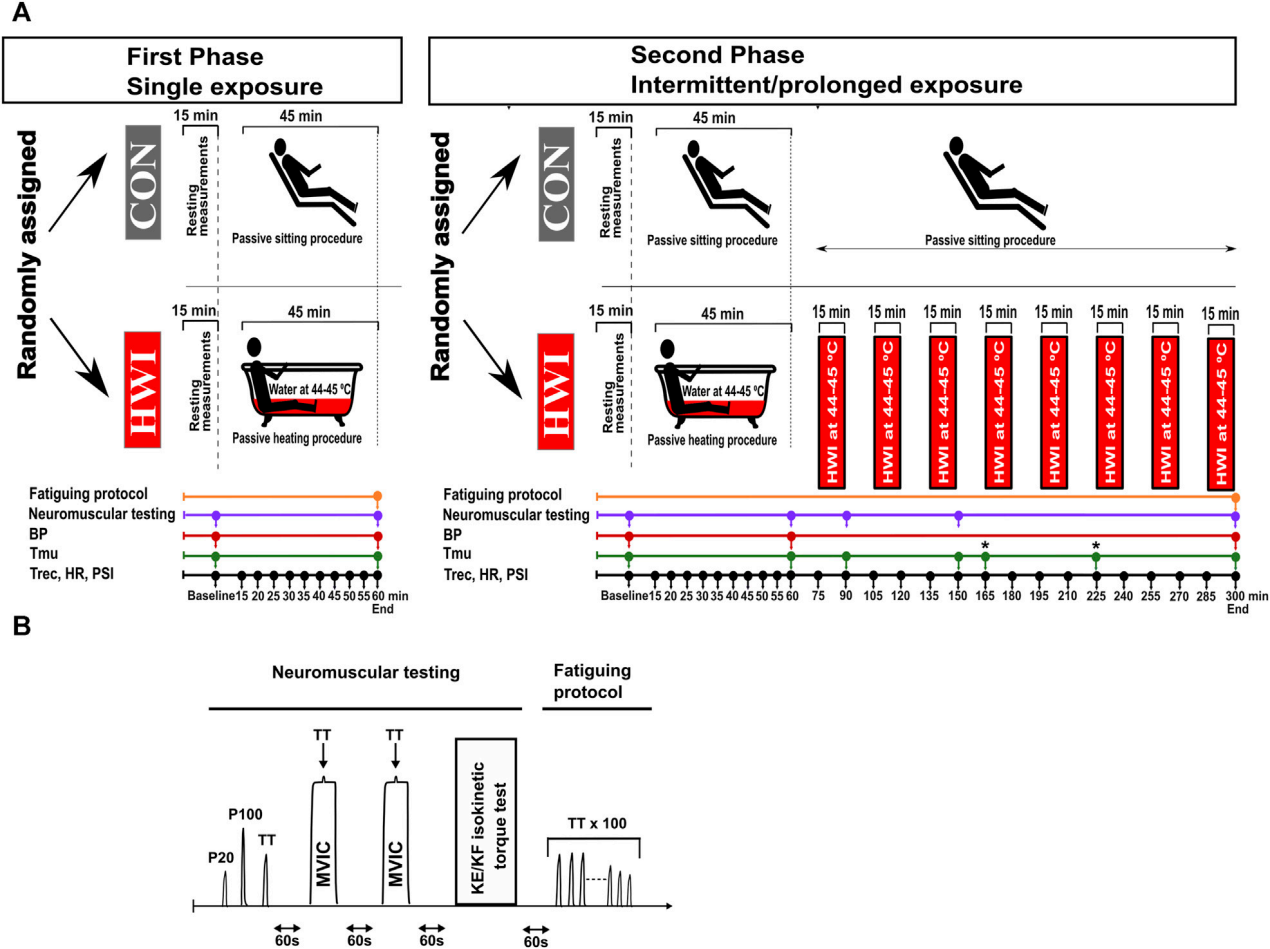
Experimental design **(A)**, and neuromuscular testing and fatiguing protocol **(B)**. BP, blood pressure; CON, control condition; HR, heart rate; HWI, hot-water immersion condition; KE, knee extension; KF, knee flexion; MVIC, maximal voluntary isometric contraction; PSI, physiological strain index; P20, electrical stimulation (1-s stimulation) at 20 Hz; P100, electrical stimulation (1-s stimulation) at 100 Hz; Tmu, intramuscular temperature; Trec, rectal temperature; TT, 250-ms test train stimulation at 100 Hz. The fatiguing protocol consisting of 100 trains of electrical stimulation of the knee extensors (100 × 250-ms TT) was only performed after the last neuromuscular testing (i.e., 2nd neuromuscular testing during the single phase, and 5th neuromuscular testing during the intermittent/prolonged phase). *: at the time points 165 and 225 min, Tmu was only measured in the HWI condition.

**FIGURE 2:**
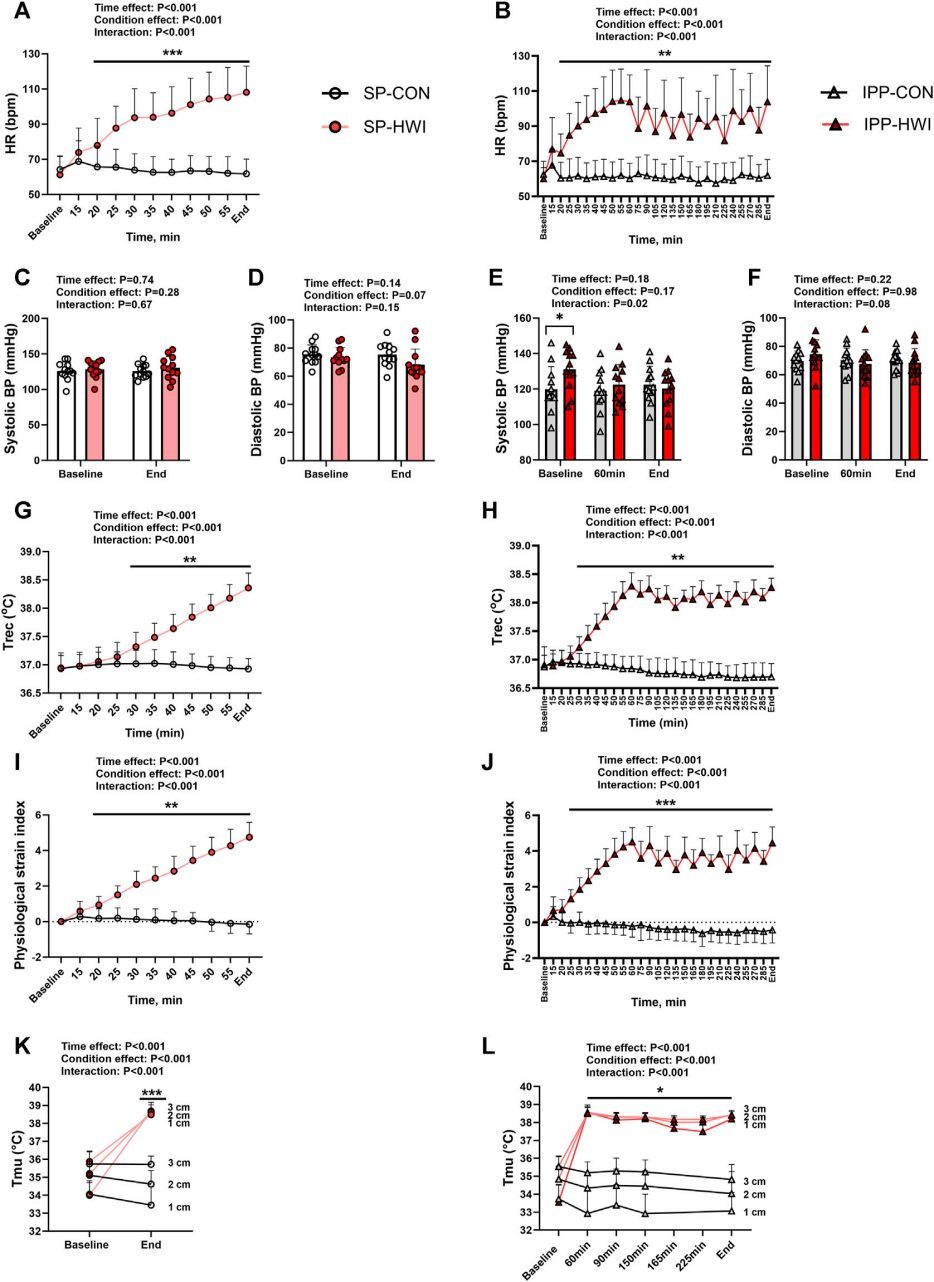
Physiological measurements during the single and intermittent/prolonged protocols. Heart rate (HR), systolic and diastolic blood pressures (BP), rectal temperature (Trec), physiological strain index (PSI) and intramuscular temperature (Tmu) at three depths (1, 2, and 3 cm) during the single **(A, C, D, G, I, K)** and intermittent/prolonged **(B, E, F, H, J, L)** protocols. Data are shown as mean ± SD and individual values are presented in panels C–F. SP, single phase; IPP, intermittent/prolonged phase. *, *p* < 0.05; **, *p* < 0.01; ***, *p* < 0.001: significant differences CON vs. HWI. In K and L, significant differences between CON and HWI are at the three depths. In L, Tmu was not assessed in CON at the time points 165 and 225 min. N = 12 for all parameters.

In this study, we developed our HWI protocols with the objective of increasing both Trec and Tmu. We used HWI as a PH method because it is practical and can induce a well-controlled heat stress (McGorm et al., 2018). During the single phase (SP-HWI), we chose the HWI protocol previously used in several studies from our research group (single bath of 45 min, 44°C–45°C, immersion up to the waist) (Brazaitis and Skurvydas, 2010; Brazaitis et al., 2011; Brazaitis et al., 2012). This protocol can substantially increase Tmu (∼2.5°C–3.0°C in the vastus lateralis muscle at a depth of 3 cm) and Trec (∼1.8°C–2.0°C) but does not lead to severe hyperthermia (i.e., Trec >39.5°C). The intermittent/prolonged phase (IPP-HWI) protocol was set by including an initial hot bath of 45 min (same protocol as for SP-HWI) followed by eight 15-min hot baths interspaced with 15-min breaks at room temperature (see Figure 1A). As shown in Figure 2, this protocol allowed to maintain elevated and relatively stable Tmu and Trec over the prolonged phase (end point at 300 min).

### 2.2 Participants

A randomized cross-over design was used in this study, in which twelve recreationally active men participated. The inclusion criteria used were: aged between 18 and 45 years, not participating in any other experiments, being healthy, physically active (at least 2–3 times per week) and without medication, and having a body mass index (BMI) < 30 kg m^-2^. The exclusion criteria were: asthma, neurological pathology, cardiovascular disease, conditions that could be worsened by exposure to heat, suffering from any kind of disease or having physical limitations that would compromise the ability to perform the neuromuscular testing, and being involved in any temperature manipulation (cold/heat acclimation) program in the 3-month period prior to the study. The age, height, body mass, percentage body fat and BMI assessed at baseline at the beginning of the study were 27.2 ± 6.6 years, 186.7 ± 7.6 cm, 86.5 ± 12.1 kg, 16.9% ± 3.1% and 24.9 ± 2.2 kg m^-2^, respectively. All experiments were performed at the Lithuanian Sports University (Kaunas, Lithuania). The study protocol was approved by Kaunas Regional Biomedical Research Ethics committee (no. P1-BE-2-14/2022) and was in agreement with the latest revision of the Declaration of Helsinki. The participants were informed of the experimental procedures and gave their written informed consent prior to participation.

### 2.3 Experimental design

The study included two phases: the phase “single exposure”, called single phase (SP), followed by the phase “intermittent/prolonged exposure”, called intermittent/prolonged phase (IPP), with both phases consisting of two experimental conditions performed in a random order: control sitting (CON) and hot-water immersion (HWI). Each experimental trial (four in total) was separated by at least 1 week. A summary of the experimental design is presented in Figure 1A. The week before the first experiment, a familiarization session was organized. During this session, the participants were familiarized with the experimental protocols and equipment, and with the different neuromuscular tests (i.e., electrically evoked contractions, maximal voluntary isometric contractions (MVIC), and maximal isokinetic concentric contractions) and the fatiguing protocol (i.e., repetitions of electrically evoked contractions). The ambient temperature in the laboratory was controlled and maintained at 23.0°C ± 0.5°C, and relative humidity was 35.0% ± 3.0%.

The participants arrived at the laboratory and started the experiments in the morning (9.00 a.m.) after overnight fasting. Body mass, body composition, heart rate (HR), blood pressure (BP), rectal temperature (Trec) and intramuscular temperature (Tmu) were assessed at baseline, (directly after sitting for 15 min). Then, they performed the first neuromuscular testing (details presented below), which was followed by either 45 min control passive sitting in the laboratory (CON condition) or by 45 min sitting in a hot-water bath (HWI condition, immersion up to the waist in an acrylic bathtub, water temperature between 44°C and 45°C). Hot water was poured into the bath, while cooler water was released to maintain the required temperature and water level. HR and Trec were recorded every 5 min during this period. Then, HR, BP, Trec and Tmu were measured, and the second neuromuscular testing was subsequently performed (endpoint during the SP and at 60 min during the IPP). During the SP, the second neuromuscular testing was directly followed by a fatiguing protocol consisting of 100 trains of electrical stimulation of the knee extensors (details presented below). During IPP-HWI, eight additional bouts of hot-water immersion (15 min each, immersion up to the waist, water temperature between 44°C and 45°C) were added after 75, 105, 135, 165, 195, 225, 255 and 285 min. In this experimental trial, participants were sitting at room temperature during the 15 min breaks between baths. During the IPP-CON trial, participants were sitting at room temperature for 300 min. During the intermittent/prolonged phase, HR and Trec were recorded every 15 min between 60 min and the end of the protocol (i.e., 300 min), and BP was measured at the end. Tmu was measured at 90 min, 150 min and at the end of CON condition (300 min), and two additional Tmu measurements were added after 165 min (before the fifth bath) and 225 min (before the seventh bath) in the IPP-HWI condition to monitor possible changes in Tmu. The number of Tmu measurements was limited to limit discomfort and tissue damage. Additional neuromuscular testing was performed at 90 min, 150 min, and at the end of the IPP. After the last neuromuscular testing, a fatiguing protocol consisting of 100 trains of electrical stimulation of the knee extensors was directly performed.

During the IPP, a strawberry breakfast cereal bar (87 kcal; Fitness, South Africa) was provided at 120 min and 240 min, while the participants remained fasted during the SP. Participants were told to take similar meals and to maintain a proper hydration on the day before experiments. During the experiments, participants did not drink water, but were allowed to rinse their mouth with cool water. Participants were allowed to go to the toilets before the beginning of the experiments, and none of them used the toilets during the experiments. They wore only swimming shorts during the baths (SP-HWI and IPP-HWI conditions), and quickly wiped off water at the end of each bath before starting the neuromuscular testing. They wore shorts and a tee-shirt in the CON conditions. In addition to these clothes, participants were wrapped in a towel between the baths in the IPP-HWI condition.

### 2.4 Physiological measurements

#### 2.4.1 Anthropometric measurements

Body mass and percentage body fat were assessed with a body composition analyzer (Tanita, TBF-300, IL, United States). The height of the participants was measured with a height gauge, and BMI was calculated. The state of hydration was estimated from body mass loss, using the following formula: body mass loss = body mass Baseline–body mass End. The mass of the two cereal bars (47 g) given during the intermittent/prolonged phase was included in the calculation.

#### 2.4.2 Blood pressure and heart rate measurements

Systolic and diastolic BP were measured on the left arm (one measurement each time) with an automatic BP monitor (Gentle+, Microlife, FL, United States). HR was recorded with a HR monitor (S-625X, Polar, Kempele, Finland). The time points of measurements are presented in the section “experimental design” and in Figure 1A.

#### 2.4.3 Body temperature measurements

Trec and Tmu were assessed, and the time points are presented in the section “experimental design” and in Figure 1A. Trec was measured using a thermocouple (Rectal Probe; Ellab, Hvidovre, Denmark; accuracy, ±0.01°C) inserted to a depth of 12 cm past the anal sphincter. Tmu was measured using a needle microprobe (Intramuscular Probe MKA, thermometer model DM-852, Ellab) inserted without anesthesia into the vastus lateralis muscle of the right leg at mid-thigh and slightly lateral to the femur at three different depths (1, 2 and 3 cm beneath the skin surface).

#### 2.4.4 Physiological strain index

The physiological strain index (PSI) was calculated using the following formula (Moran et al., 1998):

$$\text{PSI}=5\left({\text{Trec}}^{t}-{\text{Trec}}^{0}\right)\;x\;{\left(39.5\;-\;{\text{Trec}}^{0}\right)}^{-1}+5\left({\text{HR}}^{t}\;-\;{\text{HR}}^{0}\right)\;x\;{\left(180\;-\;{\text{HR}}^{0}\right)}^{-1}$$

PSI was calculated from Trec and HR, which were measured at baseline (Trec^0^ and HR^0^) and at numerous time points (Trec^t^ and HR^t^). PSI was scaled from 0 to 10, with 1–2 indicating no/little heat stress, 3–4 indicating low heat stress, 5–6 indicating moderate heat stress, 7–8 indicating high heat stress, and 9–10 indicating very high heat stress.

### 2.5 Neuromuscular testing, fatiguing protocol and data analysis

Neuromuscular testing, consisting of electrically evoked torque of the knee extensors, maximal voluntary isometric contraction (MVIC) of the knee extensors, and maximal isokinetic concentric contraction of the knee extensors (KE-isoK) and knee flexors (KF-isoK) is illustrated in Figure 1B. The participants sat upright in the seat of an isokinetic dynamometer (System 4; Biodex Medical Systems, Shirley, NY, United States) calibrated according to the manufacturer’s recommendations, with a correction for gravity performed using the Biodex Advantage program (Version 4.X). Shank, trunk and shoulders were stabilized with belts. The dynamometer was set with the knee joint positioned at an angle of 90° (180° = full extension) during MVIC and electrical stimulations, and with a joint angle ranging between 85° and 176° during dynamic voluntary contractions (KE-isoK and KF-isoK).

Electrical stimulations were applied using three 12 × 8 cm carbonized rubber surface electrodes (MARP Electronic), lubricated with electrode gel (ECG-EEG Gel, medigel, Modi’in, Israel). Two electrodes were positioned vertically and transversely across the width of the proximal portion of quadriceps muscles, and the third one covered the distal portion of the quadriceps muscles above the patella. The electrode sites were marked with a permanent marker during every trial and the participants were asked to not erase these marks between experiments. Caution was taken to similarly position the electrodes during the four experimental trials. An electrical stimulator (Digitimer DS7A, Digitimer, Hertfordshire, United Kingdom) was connected to the electrodes to deliver 0.5-ms square wave pulses at a constant current set at 100 mA and constant voltage limit set at 200 V. This selected current ensures full contraction and activation of the quadriceps muscles (Eimantas et al., 2022a).

Neuromuscular testing began with three electrical stimulations separated with 3 s of rest: 1-s stimulation at 20 Hz (P20), 1-s stimulation at 100 Hz (P100) and 250-ms test train stimulation at 100 Hz (TT). Peak torques were determined for these three electrical stimulations, and P20/P100 was calculated. The contractile properties under the unfatigued state were determined during TT by calculating contraction time/peak torque, half-relaxation time (HRT), peak rate of torque development (RTD) and peak rate of torque relaxation (RTR). Contraction time was defined as the time taken to reach peak torque. Contraction time progressively decreased in our fatiguing protocol (see the description below) as a result of the progressive reduction of torque production and thus the fact that it takes less time to reach a lower torque (data not shown). Consequently, we chose to normalize contraction time to peak torque in both protocols (i.e., unfatigued state and fatiguing protocol). This ratio represents the average time required to increase the torque by 1 Nm. HRT was calculated as the time taken for the torque to decline from the peak value to 50% of the peak value. Excel software was utilized to calculate RTD and RTR, using raw data exported from Biodex system (100 Hz sampling rate). RTD and RTR were defined as the peak slope of torque per 10 ms (Δtorque/Δ10 ms).

Directly after these three electrical stimulations (no specific warm-up was performed), two MVIC of the knee extensor muscle were performed and separated by 1-min rest. MVIC torque corresponded to the maximum torque after 2–3 s. The participants were verbally encouraged to exert and maintain maximal effort for ∼5 s, and a 250-ms test train stimulation at 100 Hz was superimposed on voluntary contraction 3–4 s into the MVIC. Central activation ratio (CAR), a measure of voluntary activation level, was calculated as: (MVIC torque/total peak torque generated with the superimposed 250-ms test train stimulation) x 100. The best attempt (with the highest MVIC torque) was selected for analysis. One minute after the second MVIC, three full repetitions of KE-isoK (90°/s) and of KF-isoK (90°/s) were performed without any interruption. The highest torque obtained over the entire range of motion from these three maximal isokinetic contractions was used for analysis.

Directly after the last neuromuscular testing (i.e., after the 2nd during the single phase and after the 5th during the intermittent/prolonged phase), a fatiguing protocol consisting of 100 trains of electrical stimulation of the knee extensor muscle (250-ms test train stimulation at 100 Hz (TT) interspaced with 1s break) was performed. TT torques and the contractile properties (contraction time/peak torque, HRT, RTD and RTR) were determined during the fatiguing protocol and values were analyzed as the average of the first three contractions, 4th to 20th contraction, 21st to 40th contraction, 41st to 60th contraction, 61st to 80th contraction, and 81st to 100th contraction (Figure 6). The torque fatigue index and the changes in contractile properties during fatiguing protocol were determined from the first three and last three contractions (Table 1).

**TABLE 1:** Torque fatigue index and changes in contractile properties during the fatiguing protocol.

		SP	IPP	Condition effect	Phase effect	Interaction
Torque fatigue index (%)	CON	54.4 ± 13.4	56.4 ± 12.4	**0.001**	0.54	0.07
	HWI	61.8 ± 10.8	58.1 ± 13.8			
Contraction time/peak torque (% change)	CON	126.4 ± 81.2	129.5 ± 90.7	**0.02**	0.98	0.85
	HWI	171.7 ± 112.2	169.5 ± 164.5			
HRT (% change)	CON	125.1 ± 81.0	121.4 ± 60.2	**0.006**	0.90	0.68
	HWI	157.1 ± 110.8	164.5 ± 85.9			
RTD (% change)	CON	−59.0 ± 16.4	−57.2 ± 15.1	**0.003**	0.35	0.86
	HWI	−68.3 ± 10.0	−67.2 ± 12.1			
RTR (% change)	CON	−77.5 ± 12.8	−76.7 ± 10.7	**0.03**	0.74	0.80
	HWI	−82.5 ± 8.3	−82.4 ± 9.1			

HRT, half-relaxation time; RTD, peak rate of torque development; RTR, peak rate of torque relaxation. Data are shown as mean ± SD, Values in bold indicate significant *p* values (*p* < 0.05). Torque fatigue index was calculated as: [(average of the first 3 contractions–average of the last 3 contractions)/average of the first 3 contractions] x 100. % change was calculated as: [(average of the last 3 contractions–average of the first 3 contractions)/average of the first 3 contractions] x 100.

N = 12 for all parameters except for RTD and RTR (N = 11).

### 2.6 Statistical analysis

Data are presented as mean ± standard deviation (SD) and data presented in figures also include individual values (except in Figure 7 and in some panels of Figure 2). Statistical analyses were performed using GraphPad Prism (Graphpad Prism 9.0.2., San Diego, CA, United States) and SPSS Statistics (version 28; for analysis of effect size only). Data were tested for normality using the Shapiro–Wilk test before conducting parametric statistical analyses, and all data were found to be normally distributed.

**FIGURE 3:**
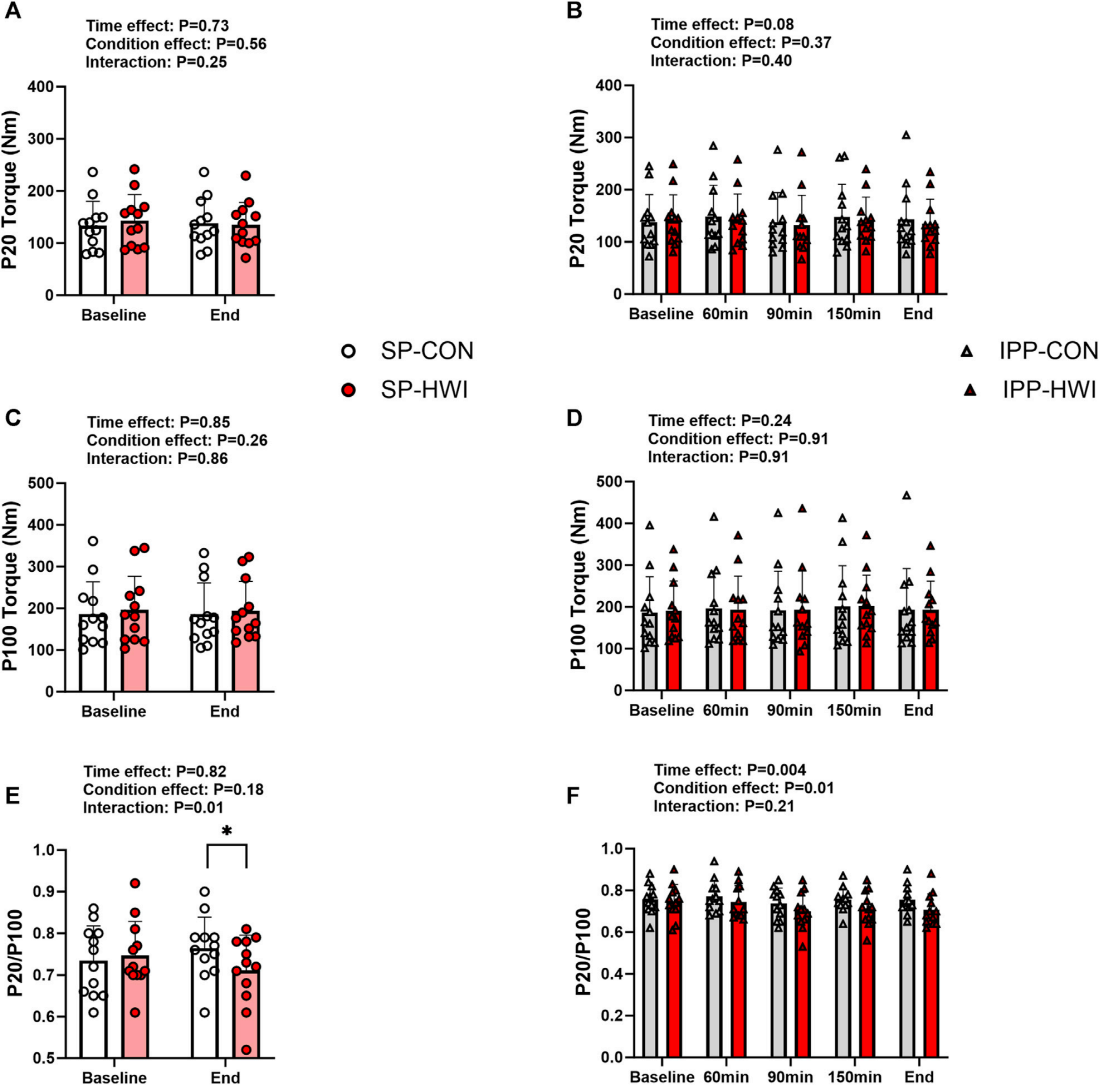
Electrically induced isometric torques under the unfatigued state. Peak torques at 20 Hz (P20) and 100 Hz (P100), and P20/P100 during the single **(A, C, E)** and intermittent/prolonged **(B, D, F)** phases. Data are shown as mean ± SD and all panels include individual values. SP, single phase; IPP, intermittent/prolonged phase. *, *p* < 0.05: significant differences CON vs. HWI. N = 12.

**FIGURE 4:**
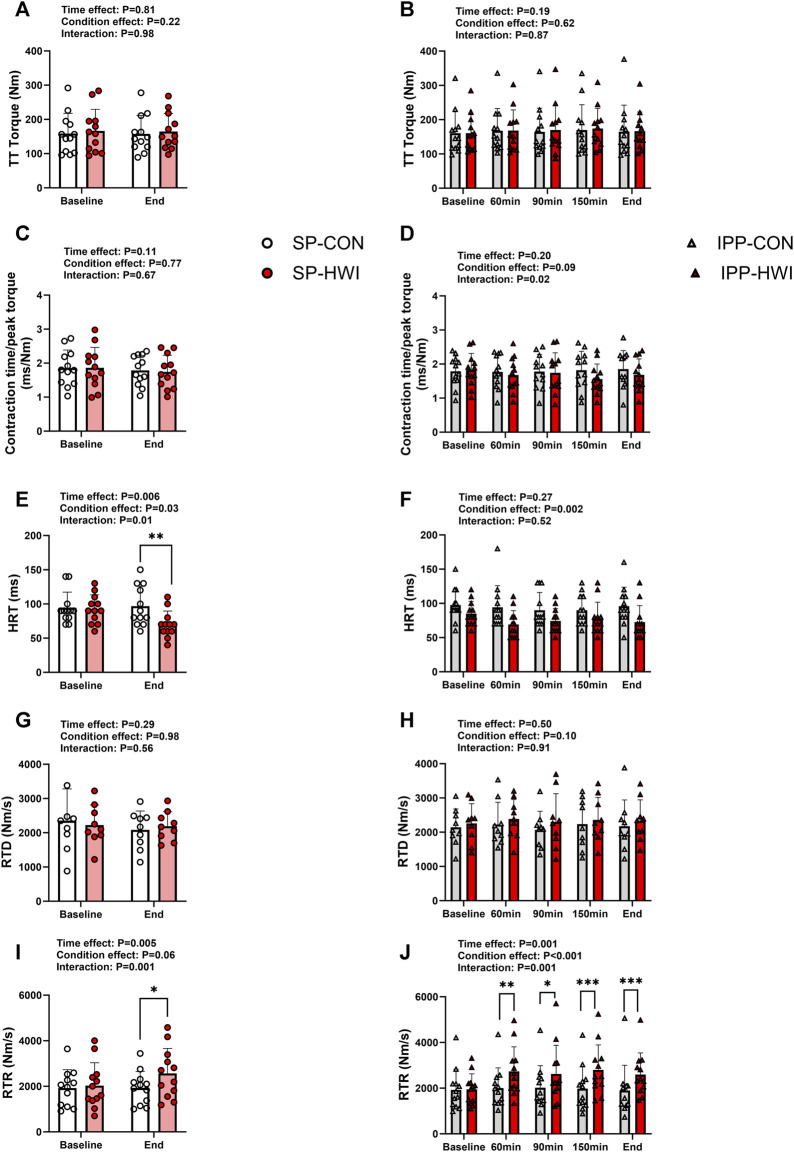
Peak torques and contractile properties derived from a 250-ms test train stimulation at 100 Hz (TT) under the unfatigued state. TT torques, contraction time/peak torque, half-relaxation time (HRT), peak rate of torque development (RTD) and peak rate of torque relaxation (RTR) during the single **(A, C, E, G, I)** and intermittent/prolonged **(B, D, F, H, J)** phases. Data are shown as mean ± SD and all panels include individual values. SP, single phase; IPP, intermittent/prolonged phase. *, *p* < 0.05; **, *p* < 0.01; ***, *p* < 0.001: significant differences CON vs. HWI. N = 12 for all parameters except for RTD (N = 9; **(G, H)**.

**FIGURE 5:**
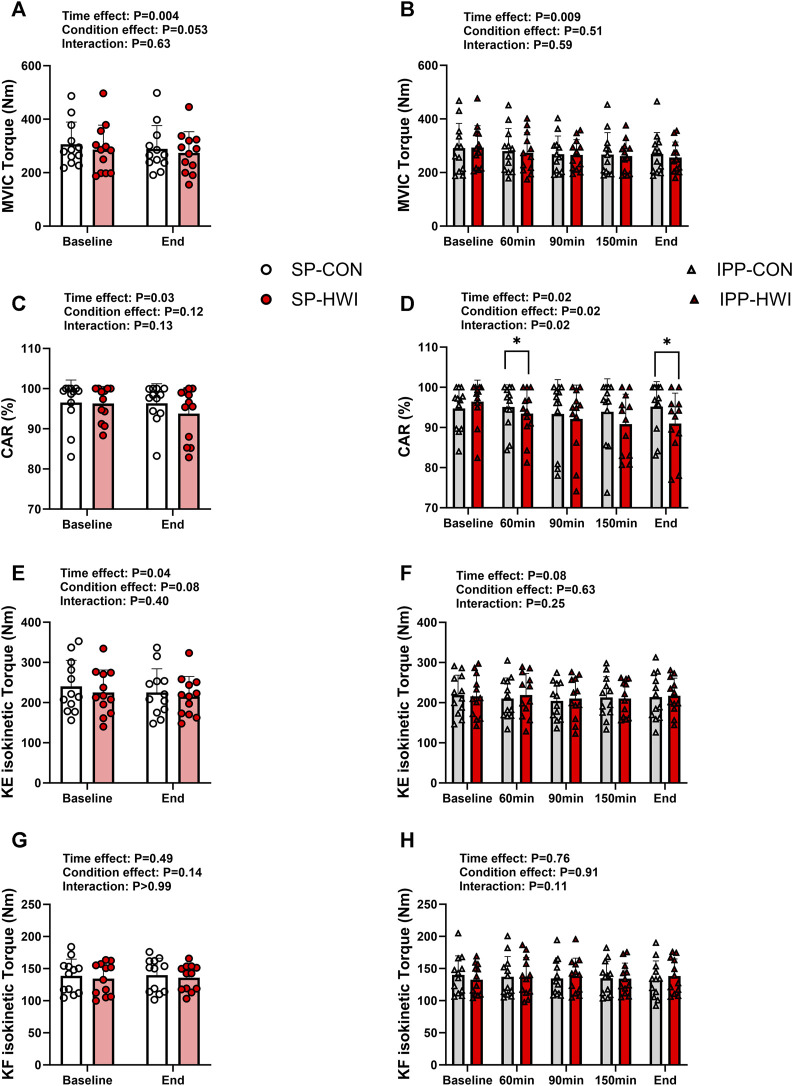
Maximal voluntary contraction torques and central activation ratio (CAR) under the unfatigued state. Maximal voluntary isometric contraction (MVIC) torques, CAR, knee extension (KE) maximal isokinetic torques, and knee flexion (KF) maximal isokinetic torques during the single **(A, C, E, G)** and intermittent/prolonged **(B, D, F, H)** phases. Data are shown as mean ± SD and all panels include individual values. SP, single phase; IPP, intermittent/prolonged phase. *, *p* < 0.05: significant differences CON vs. HWI. N = 12.

**FIGURE 6:**
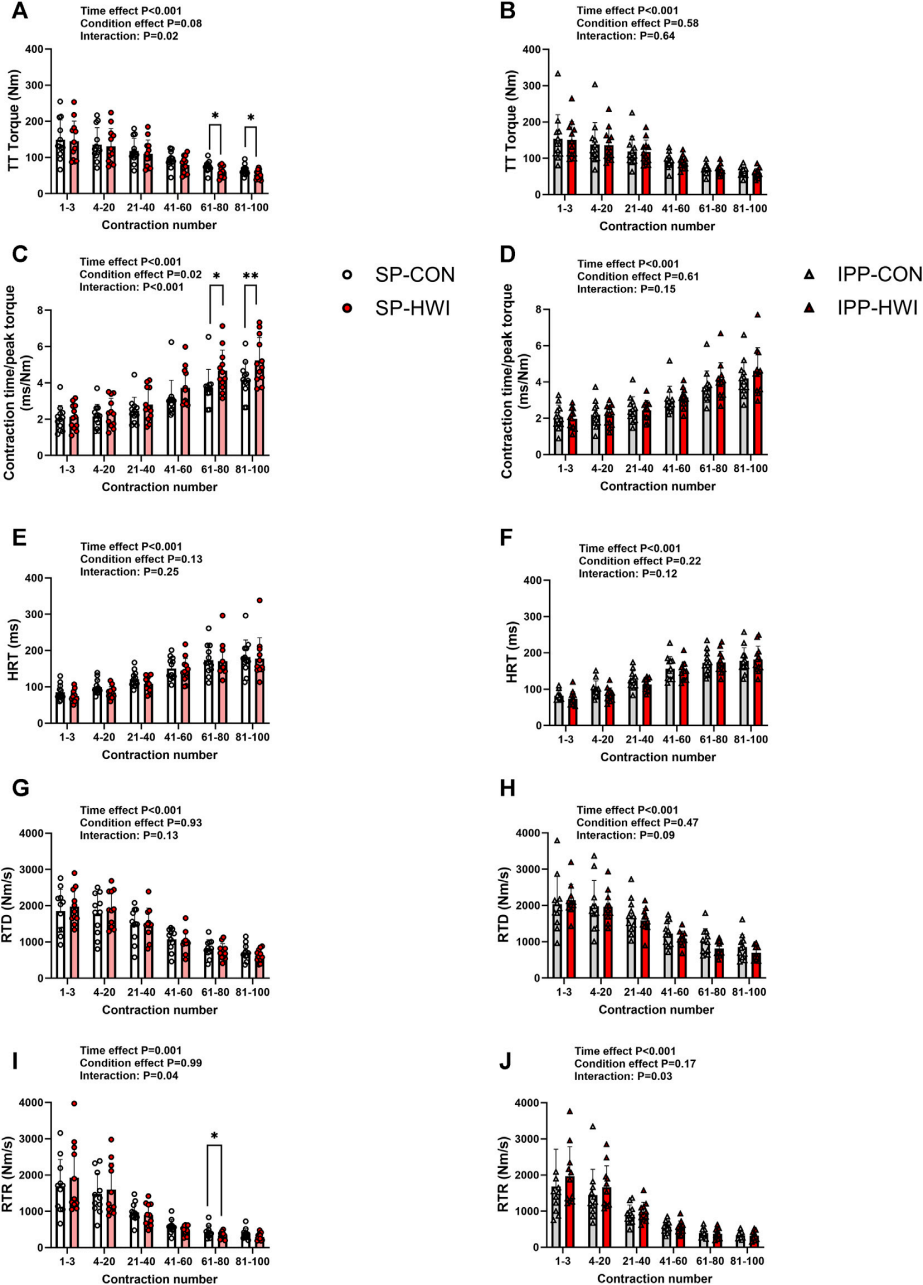
Torques and contractile properties during the fatiguing protocol. Torques during 250-ms test train stimulations at 100 Hz (TT torques), contraction time/peak torque, half-relaxation time (HRT), peak rate of torque development (RTD) and peak rate of torque relaxation (RTR) during the single **(A, C, E, G, I)** and intermittent/prolonged **(B, D, F, H, J)** phases. Data are shown as mean ± SD and all panels include individual values. *, *p* < 0.05; **, *p* < 0.01: significant differences CON vs. HWI N = 12 for all parameters except for RTD (N = 11; **(G, H)** and RTR (N = 11; **(I, J)**.

For the physiological parameters (HR, BP, Trec, physiological strain index, and Tmu presented Figure 2), and torques and contractile properties under the unfatigued state (Figures 3–5), two-way repeated-measures analyses of variance (ANOVA) were performed to assess the effects of condition (CON vs. HWI), time and the condition × time interaction during both the single and intermittent/prolonged phases. When an interaction was observed, Sidak’s multiple comparisons tests were used to compare the two conditions (CON vs. HWI). For TT torque and the contractile properties during the fatiguing protocol (Figure 6), two-way repeated-measures ANOVA were performed to assess the effects of condition (CON vs. HWI), contraction number (1–3, 4–20, 21–40, 41–60, 61–80, 81–100) and the condition x contraction number interaction during both phases. When an interaction was observed, Sidak’s multiple comparisons test were used to compare the two conditions (CON vs. HWI).

For the parameters mentioned above and for body mass loss, the Δ changes (i.e., end value–baseline value) or % changes (i.e., [(end value–baseline value)/baseline value] x 100) were calculated. These Δ changes and % changes (results only presented in the text), as well as torque fatigue index (Table 1) were analyzed using two-way repeated-measures ANOVA to assess the effects of condition (CON vs. HWI), phase (single vs. intermittent/prolonged) and the condition × phase interaction. When an interaction was observed, Sidak’s multiple comparisons tests were used to compare SP-CON vs. IPP-CON, and SP-HWI vs. IPP-HWI.

All two-way repeated-measures ANOVA included Geisser-Greenhouse corrections to correct for violation of the sphericity assumption. Three participants were excluded from the analysis of RTD under the unfatigued state, and one participant was excluded from the analysis of RTD and RTR during the fatiguing protocol due to technical issues. The α-level of significance was set at *p* < 0.05. Partial eta squared (ηp2) was determined to estimate the effect size for the two-way repeated-measures ANOVA. Cohen’s d was calculated to interpret the magnitude of the mean difference between two conditions, and effect sizes were classified as small (│d│ from 0.2 to 0.5), moderate (│d│ from 0.5 to 0.8) and large (│d│ above 0.8).

## 3 Results

### 3.1 Physiological measurements

Compared to CON conditions, HWI increased HR from 5 min of immersion (i.e., 20 min from baseline) until the end of the single and intermittent/prolonged phases (*p* < 0.01, d = 1.67 to 4.67; Figures 2A, B). During IPP-HWI, fluctuations in HR were observed from 75 min until the end of the phase as a consequence of repeated immersions to hot bath. Systolic BP remained unchanged during SP (Figure 2C), while during IPP, it was higher at baseline in HWI than CON conditions (*p* = 0.02, d = 0.99; Figure 2E). This surprising result is likely due to an anticipatory stress response preceding passive heat exposure during the IPP, as participants had already performed the SP-HWI trial at that time. Diastolic BP was not affected by experimental conditions (Figures 2D, F).

Compared to CON conditions, HWI increased Trec and PSI in the single and intermittent/prolonged phases (Trec: *p* < 0.01, d = 1.57 to 7.37, Figures 2G, H; PSI: *p* < 0.01, d = 1.60 to 7.54; Figures 2I, J). During IPP-HWI, fluctuations in Trec and PSI were observed from 75 min until the end of the phase due to the repeated hot baths. Tmu (at three different depths) increased in HWI compared to CON conditions in both SP and IPP (condition effect: *p* < 0.001, η_p_^2^ = 0.95 to 0.96; Figures 2I, J). In HWI conditions, Tmu (at three different depths) was ∼38°C–38.5°C at the end of SP and remained elevated until the end of IPP.

For all these physiological parameters, the Δ changes from baseline (i.e., end values–baseline values) were not different between SP compared to IPP in HWI conditions and in CON conditions (except Tmu at 3 cm depth. SP-CON: -0.03°C ± 0.47°C vs. IPP-CON: -0.72°C ± 0.63°C; *p* = 0.02, d = 0.90). A body mass loss was found after SP-HWI (1.16 ± 0.45 kg) and IPP-HWI (2.07 ± 0.60 kg) compared to CON conditions (condition effect: *p* < 0.001, η_p_^2^ = 0.85), with a larger loss observed after IPP-HWI than after SP-HWI (*p* < 0.001, d = 1.53). Body mass loss was also slightly higher after IPP-CON (0.41 ± 0.18 kg) than after SP-CON (0.08 ± 0.14 kg) (*p* < 0.001, d = 1.60).

### 3.2 Electrically induced torques and contractile properties under the unfatigued state

Typical 20 Hz (P20) and 100 Hz (P100) torque records are shown in Supplementary Figure S1A and results are presented in Figure 3. P20 and P100 torques were not significantly affected by the experimental conditions (Figures 3A–D). However, the ratio of P20/P100 was lower in HWI than CON conditions at the end of the single phase (*p* = 0.01, d = −0.96; Figure 3E), and over the intermittent/prolonged phase (condition effect: *p* = 0.01, η_p_^2^ = 0.44; Figure 3F). The Δ changes from baseline for P20 and P100 torques and the % changes from baseline for P20/P100 ratio were not different between SP-CON and IPP-CON and between SP-HWI and IPP-HWI.

A 250-ms test train stimulation (TT) was performed after P100 to assess the contractile properties under the unfatigued state. A typical torque record is shown in Supplementary Figure S1A. TT torques did not differ between the experimental conditions (Figures 4A, B). Contraction time/peak torque was not affected by heating during SP and IPP (Figures 4C, D). HRT was lower in HWI compared to CON at the end of single phase (*p* = 0.008, d = −1.04; Figure 4E) and over the intermittent/prolonged phase (condition effect: *p* = 0.002, η_p_^2^ = 0.61; Figure 4F). RTD was not affected by heating during SP and IPP (Figures 4G, H). RTR increased in HWI compared with CON at the end of SP (*p* = 0.01, d = 1.00; Figure 4I) and at all time points during IPP (*p* < 0.05, d = 1.09 to 2.40; Figure 4J). The Δ changes from baseline for these parameters were not different between SP-CON and IPP-CON and between SP-HWI and IPP-HWI.

### 3.3 Maximal voluntary contraction torques under the unfatigued state

MVIC torques decreased slightly during single and intermittent/prolonged phases (time effect: *p* = 0.009, η_p_^2^ = 0.54 and 0.36, respectively; Figures 5A, B), but were not significantly affected by heating. The Δ changes from baseline for MVIC were not significantly different during SP compared with IPP (phase effect: *p* = 0.09, η_p_^2^ = 0.09; SP-CON: -16.96 ± 15.84 Nm, IPP-CON: -21.38 ± 32.93 Nm, SP-HWI: -11.61 ± 28.83 Nm, IPP-HWI: -37.44 ± 39.42 Nm) and did not show any significant condition × phase interaction (*p* = 0.19, η_p_^2^ = 0.15).

CAR was affected by time in both SP and IPP (time effect: *p* < 0.05, η_p_^2^ = 0.36 and 0.27, respectively; Figures 5C, D). It was significantly lower in HWI than in CON at 60 min and at the end of the IPP (*p* < 0.05, d = −0.93 and −1.05, respectively; Figure 5D), while no time × condition interaction was found during the SP (*p* = 0.13, η_p_^2^ = 0.19; Figure 5C). Noteworthy, when including only the first two time points of the IPP protocols in the statistical analysis (to analyze the data the same way as during the SP), CAR remained significantly lower in HWI than in CON at 60 min (*p* = 0.02, d = −0.93). The % changes from baseline for CAR were larger in HWI than CON conditions (condition effect: *p* < 0.001, η_p_^2^ = 0.12; SP-CON: -0.12% ± 2.14%, IPP-CON: -0.49% ± 4.16%, SP-HWI: -2.63% ± 4.19%, IPP-HWI: -5.73% ± 4.31%), irrespective of the phase (condition × phase interaction: *p* = 0.12, η_p_^2^ = 0.20).

KE-isoK and KF-isoK torques were not affected by heating in any of the phases (Figures 5E–H). The Δ changes for KE-isoK and KF-isoK torques were not different between SP-CON and IPP-CON and between SP-HWI and IPP-HWI.

### 3.4 Electrically induced torques and contractile properties during fatiguing protocol

**FIGURE 7:**
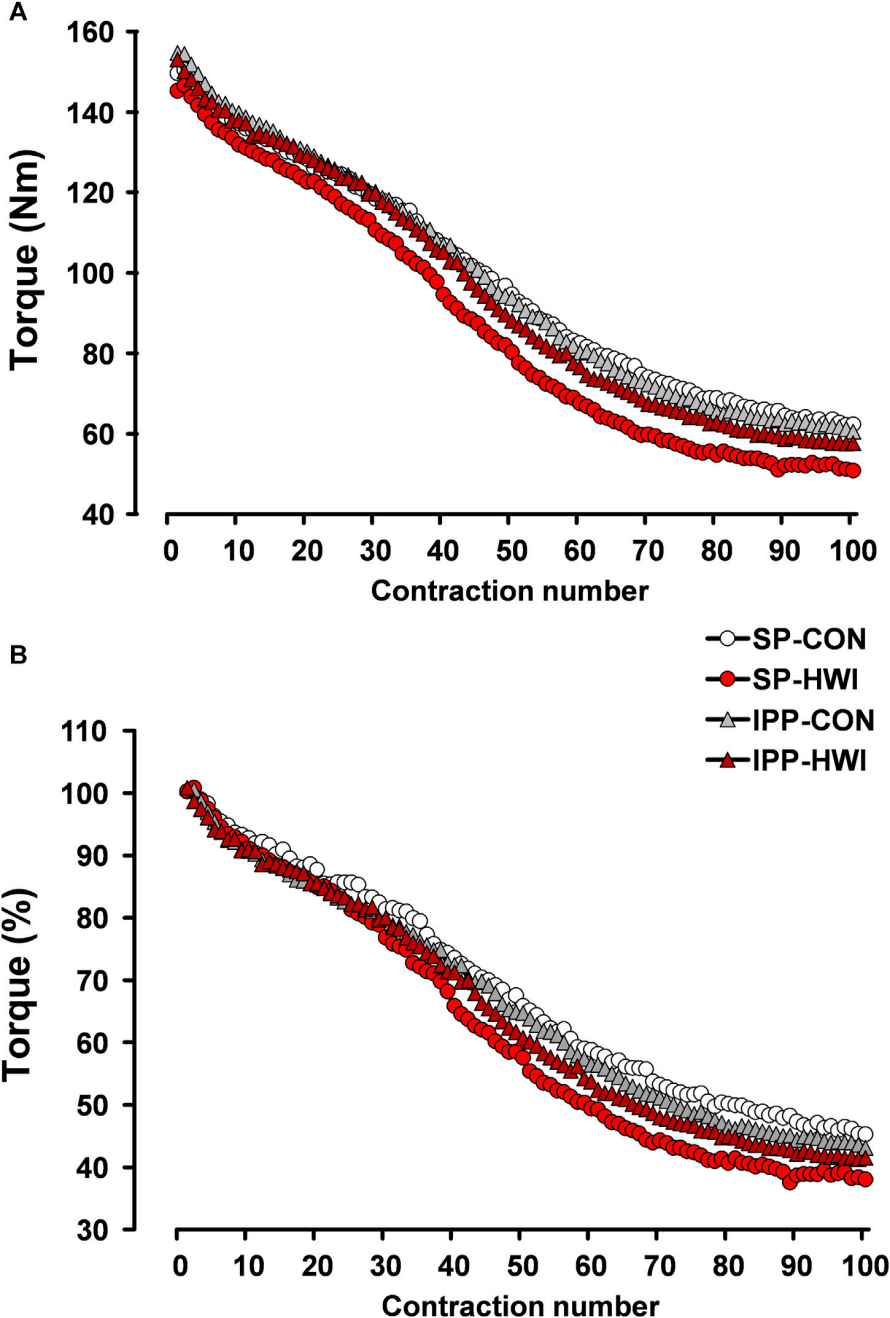
Peak torques during the fatiguing protocol, expressed in Nm **(A)** and in percentage **(B)**. Data are shown as the means obtained from the 12 participants.

A fatiguing protocol, consisting of 100 electrical stimulations of the knee extensor muscle [250-ms test train stimulation at 100 Hz (TT) interspaced with 1-s break], was performed at the end of the single (i.e., at 60 min) and intermittent/prolonged (i.e., 300 min) phases. TT torques and the contractile properties are presented in Figures 6, 7. Typical torque records are illustrated in Supplementary Figure S1B. Decreases in TT torques, RTD and RTR, and increases in contraction time/peak torque ratio and HRT were found over time in CON and HWI conditions for both phases, as illustrated in Figure 6 (time effect: *p* < 0.001, η_p_^2^ = 0.71–0.85) and Figure 7. After the single phase, significant time × condition interactions were found for TT torques, contraction time/peak torque ratio and RTR (*p* < 0.05, η_p_^2^ = 0.34 to 0.59; Figures 6A, C, I) but not for HRT and RTD (Figures 6E, G). For this phase, post-hoc analyses detected higher contraction time/peak torque ratio during the last 40 contractions in HWI compared to CON (*p* < 0.05, d = 1.16 to 1.25; Figure 6C). In addition, lower TT torques were found during the last 40 contractions and lower RTR were observed between the 61st and 80th contractions in HWI compared with CON (*p* < 0.05, d = −1.01 to −1.07; Figures 6A, I). After IPP phase, no time × condition interactions were found for any of these parameters, except for RTR (*p* = 0.03, η_p_^2^ = 0.36; Figure 6J). However, post-hoc analysis did not reveal any differences between HWI and CON conditions.

Torque fatigue index, the increases in contraction time/peak torque ratio and in HRT, and the decreases in RTD and in RTR were larger after HWI conditions (condition effect: *p* < 0.05; η_p_^2^ = 0.39 to 0.63; Table 1). No significant condition × phase interactions were found for any of these parameters. The differences of torque fatigue index between the experimental conditions are illustrated in Figure 7.

## 4 Discussion

The present study investigated the impact of 1) PH induced by single and intermittent/prolonged HWI, and 2) the duration of PH, on muscle force production and contractile properties under the unfatigued state and during repeated electrically evoked contractions in young males. Under the unfatigued state, PH did not significantly affect P20, P100 and TT torques, but it induced a shift towards a faster contractile profile, as evidenced by a decreased P20/P100 ratio and an improved muscle relaxation (i.e., reduced HRT and increased RTR). These results were associated with an elevated Tmu and were independent of the duration of heating. Despite an increased Trec and a reduced voluntary activation, PH did not impair MVIC torques, and KE-isoK and KF-isoK torques remained unaffected. During the fatiguing protocol, torque fatigue index and the changes in muscle contractile properties were larger in HWI than in CON conditions. However, these parameters were not significantly affected by the duration of heating.

### 4.1 Involuntary contractions under the unfatigued state

Our results showed that P20, P100 and TT torques were not affected by PH, despite a substantial elevation of Tmu. These findings are in agreement with previous studies which determined these parameters in young and healthy males (Davies et al., 1982; Brazaitis et al., 2019; Baranauskiene et al., 2023). This supports the idea that elevated intramuscular temperature does not influence tetanic force during electrically evoked contractions, at least in our experimental setting. Furthermore, we observed that PH induced a shift in contractile characteristic towards a faster phenotype, as evidenced by a decreased P20/P100 ratio and an improved muscle relaxation (i.e., reduced HRT and increased RTR). PH preferably improved muscle relaxation without affecting muscle contraction velocity (i.e., contraction time/peak torque and RTD). This finding confirms some previous observations (Todd et al., 2005; Periard et al., 2014b), but is inconsistent with others (Davies et al., 1982; Davies and Young, 1983; Racinais et al., 2017; Brazaitis et al., 2019; Rodrigues et al., 2021). The discrepancy between these studies may relate to differences in muscle characteristics (knee extensors and ankle plantar flexors), passive heating protocols (type, duration of heat exposure, temperature, etc.), and neuromuscular testing (dynamometer, type of contraction, joint angle, etc.). Furthermore, and in accordance with our hypothesis, the changes in muscle contractile properties induced by PH remained similar after SP-HWI and IPP-HWI due to the identical elevation of Tmu. This demonstrates that intramuscular temperature (and potentially the temperature of tendons and other integral connective tissue) (Mornas et al., 2022; Rodrigues et al., 2022) rather than the duration of PH, is the primary factor determining muscle contractile properties under the unfatigued state.

### 4.2 Maximal voluntary contractions under the unfatigued state

Previous studies showed that PH reduced MVIC force, a result accompanied with a decrement in voluntary activation and an increased Trec (Morrison et al., 2004; Thomas et al., 2006; Periard et al., 2014a), indicating a hyperthermia-induced central fatigue. In contrast to these studies where the elevation of Trec was >2°C, Trec only increased by ∼ 1.4°C with SP-HWI and IPP-HWI. This lower elevation of Trec was able to slightly decrease voluntary activation (approximately −3% after SP-HWI and −6% after IPP-HWI; condition effect: *p* < 0.001), but it was not sufficient to significantly impair MVIC torque, a result consistent with a recent study (Mornas et al., 2022). An absence of reduced MVIC force was also reported in the knee extensor muscles during moderate hyperthermia induced by PH (Trec increase of ∼1.4°C) (Periard et al., 2014a), while MVIC torque was impaired in response to a similar increased Trec (∼1.3°C) in the elbow flexor muscles (Todd et al., 2005). This suggests that the impact of moderate hyperthermia on MVIC force could depend on the muscle characteristics and might be more deleterious in smaller muscle groups. Furthermore, other studies using a similar protocol as SP-HWI protocol (45 min at 44°C) did not find any decline of MVIC torque of the knee extensors (Stanley et al., 1994; Brazaitis et al., 2011). Core temperature was not reported in one study (Stanley et al., 1994), while the increase in Trec was ∼1.8°C in the other one (Brazaitis et al., 2011). The discrepancy in thermal response between the latter study and our work may come from anthropometric differences between the participants, with the young males recruited in our study being ∼7.5 kg heavier. Finally, a severe elevation of core temperature (>2°C) led to a reduction of the MVIC force produced by the knee extensor muscles during prolonged contractions (≥5 s) (Morrison et al., 2004; Periard et al., 2014a), but not during brief contractions (2 s) (Nybo and Nielsen, 2001). As reported above, MVIC torque was not impaired in response to an elevated Trec of 1.8°C (Brazaitis et al., 2011), but this was found for rather short isometric contractions (3 s). Altogether, it is conceivable that PH impairs MVIC force generated by the knee extensor muscles when the elevation of core temperature is >2°C (and Trec >39.5°C) and/or when maximal contraction is sustained for >5 s.

It has been recently shown that MVIC torque of the knee extensors and voluntary activation were reduced after prolonged HWI (90 min at 42°C) (Rodrigues et al., 2023). Compared to shorter elevation of Trec (i.e., SP-HWI), our results showed that prolonged elevation of Trec (i.e., IPP-HWI) did not exacerbate MVIC force depression (assessed from the Δ changes) and CAR reduction (assessed from the % changes). The elevation of Trec (∼1.4°C) and PSI (∼4.5–4.8) were similar directly after SP-HWI and IPP-HWI and represent a relatively moderate heat stress, at least when compared with that observed by Morrison et al. in response to PH (Trec increase of 2.1°C and PSI of ∼8) (Morrison et al., 2004). It is possible that a severe heat stress (i.e., Trec increase >2°C) for several hours would aggravate PH-induced MVIC force reduction due to central drive inhibition. Future experiments cautiously designed to avoid medical complications such as heat stroke could be relevant to test this hypothesis.

Our results showed that KE-isoK and KF-isoK torques assessed at moderate velocity (90°/s) were not affected by PH. This finding is consistent with a previous study using a similar HWI protocol (45 min at 44°C) (Stanley et al., 1994), in which KE-isoK force was determined in a wide range of velocities (30°–400°/s). Since peak torque is only attained at a specific joint angle during isokinetic contractions, an improved capacity to quicky generate force would increase peak torque during such contractions. This was not the case in our study, as evidenced by an absence of effect of PH on RTD and contraction time/peak torque during involuntary contractions. In contrast, Sargeant observed an improvement of isokinetic peak force during 20 s maximal cycling sprints performed after PH (HWI for 45 min at 44°C) (Sargeant, 1987), which was larger at high (140 revolutions.min^-1^) than low (54 revolutions.min^-1^) pedaling rates. The controversy between the latter results and those of Stanley et al. and ours probably comes from the characteristics of the isokinetic exercises (cycling vs. knee extension, multi-joint vs. single-joint exercises).

### 4.3 Muscle contractile function during the development of fatigue

Numerous studies have demonstrated that PH impairs fatigue resistance during sustained MVIC (Morrison et al., 2004; Todd et al., 2005; Brazaitis et al., 2012; Periard et al., 2014a; Baranauskiene et al., 2023), due to both central (to a large extent) and peripheral (to a lower extent) mechanisms. To our knowledge, only one previous study investigated the impact of PH on muscle fatigability during repeated electrical stimulations (Brazaitis et al., 2011). In accordance with this study, we observed that HWI-induced increase in Tmu was associated with a larger decline of force. This was however less evident here than in the previous study, probably because the overall drop of force (when all conditions were considered) during the development of fatigue was more pronounced in the previous work (∼ 85% drop) than in the current work (∼ 60%). This is likely explained by the specificity of the fatiguing protocol used (50 x 1-s TT at 50 Hz interspaced with 0.4 s break in Brazaitis’s study vs. 100 × 250-ms TT at 100 Hz interspaced with 1 s break in the current study). In addition, we observed that compared to the CON conditions, HWI induced larger changes in muscle contractile properties (contraction time/peak torque, HRT, RTD and RTR), which aligns with the impaired fatigue resistance observed during the repeated contractions. Our analysis of muscle contractile properties under the unfatigued state indicated that increased Tmu preferably accelerated muscle relaxation, which could be due to increased rates of Ca^2+^ reuptake and cross-bridge detachment (Rodrigues et al., 2022). This could increase the rate of ATP consumption and energy cost (Ball, 2021), which ultimately would lead to the larger fatigability observed during electrically induced contractions.

In contrast to our hypothesis, prolonged elevation of Tmu did not exacerbate the decline of torque during the development of fatigue. This result indicates that muscle fatigability is mainly determined by intramuscular temperature rather than the duration of heat exposure during electrically induced contraction.

### 4.4 Limitations

HWI was used as a PH method to increase muscle and rectal temperatures, and the study was performed in young and recreationally active males. Therefore, our findings may not be replicable using other heating methods and in other populations (e.g., females, elite athletes, etc.). Future research would be of interest to investigate these aspects. HWI is a practical heating method that induces a well-controlled heat stress (McGorm et al., 2018). However, IPP-HWI was perceived as challenging by our young and healthy participants, suggesting that it may not be easily applicable in real-life settings. Interventions including HWI may be relevant in clinical populations with low functional capacity to improve muscle contractile function (Rodrigues et al., 2023). Due to safety issues and the lack of additional beneficial effects of IPP-HWI (vs. single HWI), this type of heating method would not be recommended in clinical populations with higher risks for heat-related illness (e.g., elderly, and obese populations) (Speakman, 2018; Millyard et al., 2020). Finally, our control condition consisted of passive sitting, a condition used in most of the studies using HWI. Others have used thermoneutral water immersion (36°C) as an alternative control condition (Rodrigues et al., 2021; Rodrigues et al., 2023). This approach would have the advantage of maintaining similar hydrostatic pressure and movements (i.e., stepping in and out of the bath on numerous occasions) between conditions. Because thermoneutral water immersion at 36°C for 90 min slightly increases Tmu, a lower water temperature (34 or 35°C) would be more appropriate (Rodrigues et al., 2021; Rodrigues et al., 2023).

### 4.5 Practical implications and perspectives

Although PH does not increase force production during involuntary tetanic contractions, and voluntary isometric and isokinetic contractions, ours results indicate that increased Tmu enhances muscle contractile properties (especially muscle relaxation) and induces a shift towards a faster contractile phenotype. These Tmu-mediated changes in muscle function may be beneficial for acute explosive exercises (Asmussen et al., 1976; Bergh and Ekblom, 1979). However, elevated Tmu impairs fatigue resistance during repeated involuntary contractions, suggesting that passive muscle heating could increase muscle force decline during repetitive explosive exercises and intense cyclic exercises. In addition, our results clearly demonstrate that passive heating-induced changes in muscle contractile function are not further augmented by prolonged exposure (i.e., up to 285 min) when heat stress is moderate, indicating that prolonged heating is not beneficial for this purpose. Further research is required to investigate whether these changes are influenced by the duration of exposure to severe heat stress. This is of practical relevance for healthy populations (e.g., athletes, soldiers, and individuals in different occupational and recreational settings) exposed to hot and humid conditions for prolonged durations.

### 4.6 Conclusion

Our results provide clear evidence that under the unfatigued state, increased Tmu improves muscle contractile properties (especially muscle relaxation) and induces a shift towards a faster contractile profile, regardless of the duration of heating. Moreover, passive heating does not impair muscle force during maximal voluntary isokinetic concentric contraction and during maximal voluntary isometric contraction, despite a moderate reduction of voluntary activation (approximately −3% after SP-HWI and −6% after IPP-HWI). Finally, elevated Tmu increases muscle fatigability during electrically induced contractions, which is not aggravated by prolonged muscle heating. Altogether, our findings indicate that passive heating-induced changes in muscle contractile function are not further augmented by prolonged exposure in young males experiencing moderate thermal stress.

## Data Availability

The raw data supporting the conclusion of this article will be made available by the authors, without undue reservation.
