# Supplementary figures and images for: Passive heating-induced changes in muscle contractile function are not further augmented by prolonged exposure in young males experiencing moderate thermal stress
Source: Front Physiol. 2024 Feb 27;15:1356488. doi: 10.3389/fphys.2024.1356488 (PMC10928533; doi:10.3389/fphys.2024.1356488)

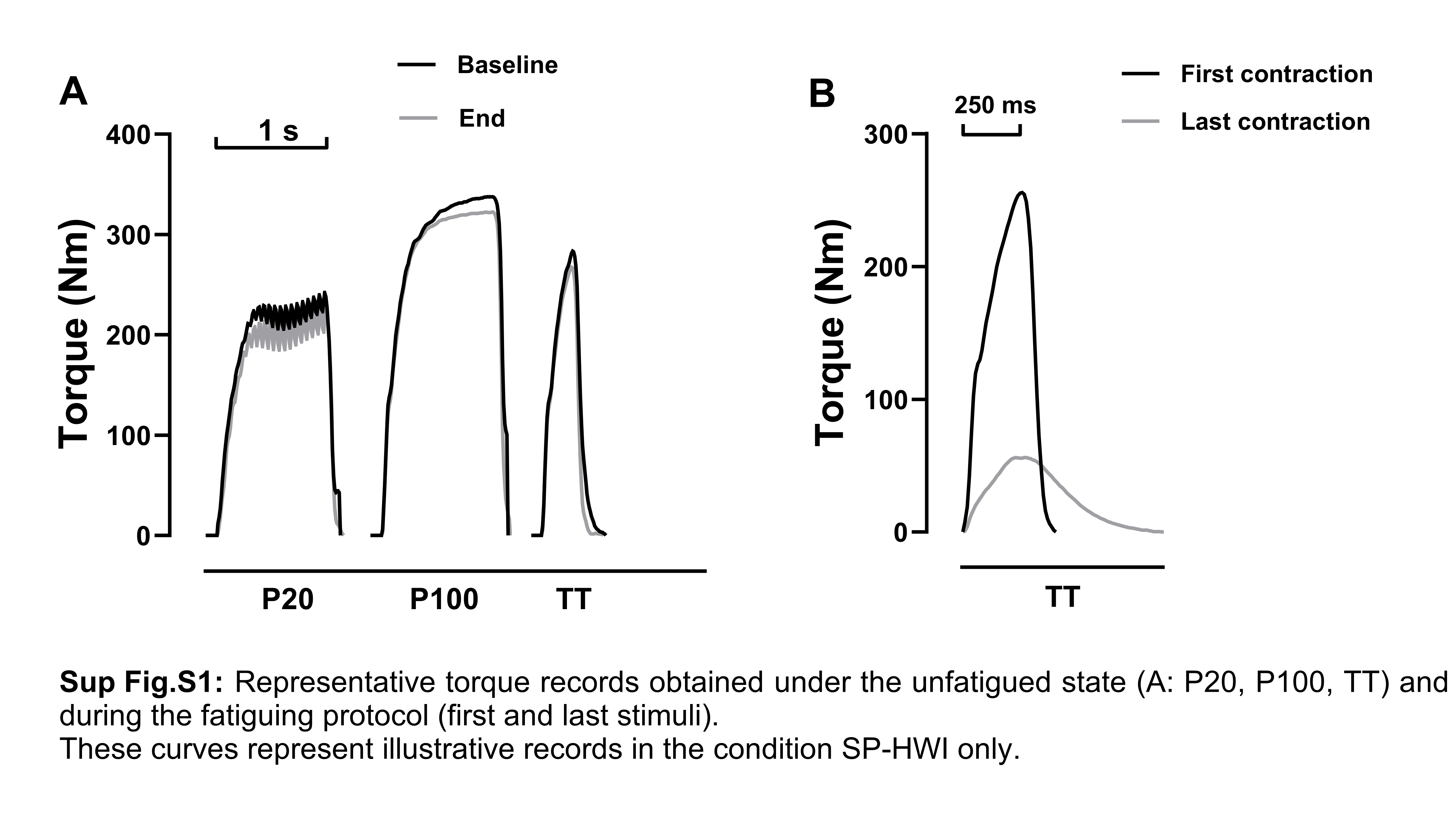

Supplement: Supplementary file 1 [file Image1.tif]
